# Data-Driven Supply Chain Operations—The Pilot Case of Postal Logistics and the Cross-Border Optimization Potential

**DOI:** 10.3390/s23031624

**Published:** 2023-02-02

**Authors:** Tanja Zdolsek Draksler, Miha Cimperman, Matevž Obrecht

**Affiliations:** 1 Jožef Stefan Institute, Jamova Cesta 39, 1000 Ljubljana, Slovenia; 2 Faculty of Logistics, University of Maribor, Mariborska Cesta 7, 3000 Celje, Slovenia

**Keywords:** cognitive logistics, machine learning, social IoT, last mile, digital twin, supply chain digitization

## Abstract

According to the defined challenge of cross-border delivery, a pilot experiment based on the integration of new digital technologies to assess process optimization potential in the postal sector was designed. The specifics were investigated with events processing based on digital representation. Different events were simulated with scenario analysis with the integration of the Cognitive Advisor and supported by the monitoring of KPIs. The business environment is forcing logistics companies to optimize their delivery processes, integrate new technologies, improve their performance metrics, and move towards Logistics 4.0. Their main goals are to simultaneously reduce costs, environmental impact, delivery times, and route length, as well as to increase customer satisfaction. This pilot experiment demonstrates the integration of new digital technologies for process optimization in real time to manage intraday changes. Postal operators can increase flexibility, introduce new services, improve utilization by up to 50%, and reduce costs and route length by 12.21%. The Cognitive Advisor has shown great potential for the future of logistics by enabling a dynamic approach to managing supply chain disruptions using sophisticated data analytics for process optimization based on the existing delivery infrastructure and improving business processes. Research originality is identified with a novel approach of real-time simulation based on the integration of the Cognitive Advisor in postal delivery.

## 1. Introduction

### 1.1. Emerging Trends in Postal Logistics

The observed trends and developments in the postal sector that are emerging for the mid- and long-term future are mostly related to the development and application of new technologies. Digitization and automation, as well as innovations in terminal devices (e.g., smartphones and tablets) and the abundance of communication options, are influencing consumer behavior and business practices, leading to platformization, e-substitution, promotion of e-commerce, and innovations in delivery methods as well as supply chain resilience [1]. Changing user needs and preferences and technological developments are changing perceptions of postal markets and services. In addition, the trade-offs between electronic and physical services are becoming more intertwined. Postal operators are benefiting from technological advances to develop new business models and innovative services that can also benefit consumers (e.g., live tracking and same-day delivery) [2,3].

According to some studies, Industry 4.0 and the new paradigm of simulation modeling enable multiple benefits in supply chain optimization [4,5]. Transportation for process innovation and distribution for product innovation are rated as the two most promising parts of postal innovation at the core of supply chains [6].

The process of delivery is one of the biggest challenges to supply chain reliability and efficiency. The existing methods, systems, and tools do not provide detailed monitoring of this process. Therefore, the need for an alternative to monitor and manage delivery has been identified, as current methods are not able to accurately assess where intervention is required or unnecessary. Artificial intelligence/machine learning (AI/ML)-based solutions are promising in their ability to (1) distinguish cases where intervention is needed and (2) manage interventions more efficiently and in real time [7,8]. 

Digital transformation technologies have a major impact on the way organizations are transforming [9]. Analytical tools and applications have been developed to analyze and extract value from the large amounts of data available to businesses. Cyber-physical model-based solutions should first be tested on digital twin simulations that integrate real-time data [5], which can outperform traditional data management and decision-making based on insufficient past data and the uncertainty of human behavior in complex situations of a relatively simple postal distribution. Upgrading to real-time tracking is beneficial to provide more efficient decision support and transparent insight into a supply chain consisting of multiple parties, especially when multiple vendors and outsourced logistics providers are involved [10].

This article highlights the current state of the art (AI/ML, Big Data analytics, social Internet of Things (social IoT)) in cross-border postal logistics. It leverages existing infrastructure to develop new business models. The goal is to leverage existing data, data technologies, and infrastructure to process delivery events in real time. A pilot experiment is presented that focuses on the transition to data-driven operations and real-time information management based on a simulation of the digital twin of cross-border postal distribution.

### 1.2. Advances of the Postal Industry Digitalization

Postal services are services of general economic interest that play an essential role in achieving an effective single market for the EU, but the traditional postal paradigm has to transform into more efficient and digitized systems [11]. The postal sector is also a key player in promoting socioeconomic development, reducing transaction costs among economic actors, and enabling access to an extensive communications and infrastructure network, which consequently contributes to achieving the United Nations Sustainable Development Goals (SDGs) [12].

However, there are significant differences among postal service providers. The Postal Development Report [12,13] ranks Switzerland (100 points), Austria (95.35 points), and Germany (94.22 points) as top performers. The Integrated Index for Postal Development shows that the development gap in postal services is significant. Post of Slovenia, representing a pilot in this study, ranks 40th (56.16 points). All leading postal operators are characterized by high reliability, reach, and resilience, which can be further improved by modeling the digital twin for optimized postal distribution toward the direction of future postal development. This is especially true for small postal operators, allowing for greater flexibility and rapid implementation of promising new technologies and business models [14,15].

In addition to postal development opportunities for e-commerce, the postal service is increasing its de facto reliance on a highly competitive segment where reliability and reach are prerequisites for success. In such an environment, the only way for postal operators to increase their relevance in the long term is to attract and retain customers who order goods online and value timeliness and predictability in delivering domestic or imported (global) products. This underscores the importance of international cooperation in areas such as knowledge sharing, common standards, and the integration of new technologies. Most parcel deliveries are handled by universal postal service providers, but delivery by private operators is spreading rapidly [6]. At this point, companies need to take appropriate measures to reconcile what digital transformation means for them in terms of economic impact [16].

A key challenge, especially for optimizing cross-border distribution and multi-stakeholder delivery, is to ensure the quality of physical delivery of goods ordered online using innovative additional value-added services. To ensure competitiveness and a continuous increase in postal services efficiency, new nontraditional activities need to be explored and tested to unlock new savings potential [11,17]. The digital technologies addressed in this study enable the optimization and digitization of tomorrow’s postal distribution processes with real-time responses [18].

### 1.3. Gap, Scope, and Objectives

In the literature reviewed, most studies on delivery optimization focus on fleet optimization, new infrastructures, and numerical simulations [8] or propose or compare different existing optimization tools and methods [19]. Since some already integrate real-time data [20], or even real-time data and digital twin simulations, but with different and somewhat distant goals for distribution companies (e.g., staff efficiency [5] or even medical complications [21]), there is a lack of studies that address the development of a new business model that leverages existing data and infrastructure but is supported by AI/ML to optimize delivery events and manage delivery as a process in real time.

Due to the availability of data and AI/ML technology, as well as the social IoT, this paper is oriented around a pilot experiment based on the simulation of the digital twin for the challenges of cross-border postal delivery. Postal operators are not yet fully digitalized and are still manually planning and performing many operations. The focus is on developing a new business model based on precise process optimization for dynamic real-time postal Logistics 4.0. Section 2 describes the pilot experiment challenge, objectives, digital representation, scenarios, and key performance indicators (KPIs) studied. Section 3 presents results focused on mitigation of a quick supply chain and distribution disruptions, considering practical challenges such as vehicle breakdowns, traffic accidents, etc., and changes in the designed and monitored logistical KPIs. In Section 4, the developed solution and performance are discussed.

## 2. Pilot Experiment & Methodology

### 2.1. Challenge Definition

The goal of the cross-border pilot was to optimize route and delivery planning between two postal operators (Post of Slovenia and Post of Croatia). The most important factors for optimization and planning processes are daily parcel flows supported by a specific fleet portfolio. Postal operators are not yet fully digitalized and perform many operations manually. This pilot experiment brings new insights into digitalized processes and deliveries. In the case of cross-border parcel delivery, order management was optimized by assigning the right order to the right vehicle and deciding whether a vehicle needed to be diverted. Since Post of Slovenia is relatively small (Slovenia: population approx. 2 million people), compared to other postal operators, and has a high number of cross-border deliveries, it is well suited as a pilot case for simulating cross-border parcel delivery.

**Figure 1:**
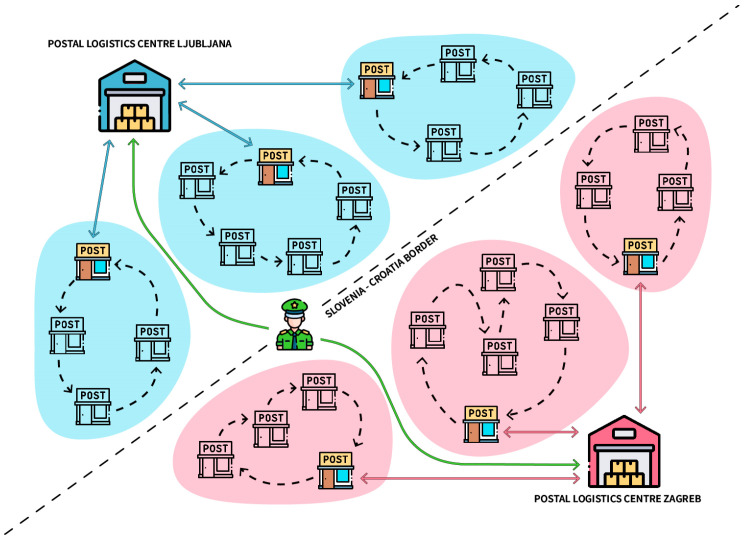
Existing parcel flow in a cross-border region.

Figure 1 shows Slovenia (blue) and Croatia (red) with the current baseline parcel flow. Parcels always arrive for nationwide delivery, regardless of the recipient’s location. They arrive from the foreign postal logistics center (office of exchange) and are received and scanned at the domestic postal logistics center (office of exchange) (green line). Parcels are then sorted and prepared for transport (blue line) to the delivery post office. From the delivery post office, parcels are collected and distributed (black line) between other post offices and the private addresses of the recipients. At the same time, the parcels are collected from the post offices and returned to the delivery post office, from where they are sent to the domestic postal logistics center of exchange and then forwarded to the foreign one, and the cycle starts all over again. It is not possible for the parcels to deviate from the established routes. Tracking is only possible through the prescribed tracking events (called EMS events) of the Universal Postal Union (UPU), which are not in real time, and there are also not enough scans to track the parcel accurately.

With the existing workflow, there is little to no flexibility to manage intraday changes in the delivery infrastructure. New events that occur at the level of parcel requests can be processed the next day, resulting in a response time of one day. In addition, developing new services, such as dynamic changes in the delivery address for parcels, is impossible. Moreover, the parcel delivery process is controllable only during initial route planning, which is also far from optimal. 

According to the defined challenge of cross-border delivery, the pilot’s objectives were first defined (Section 2.2). A model of cross-border postal parcel delivery was created as a digital twin (Section 2.3), and different events were simulated with four test scenarios (daily plan, ad hoc, traffic disruption, cross-border event) (Section 2.4). The simulation was performed using Open Stream Map (OSM) and the CA system (Section 2.5). The background of the measured KPIs is explained in Section 2.6.

### 2.2. Objectives and the Pilot Experiment Design

**Figure 2:**
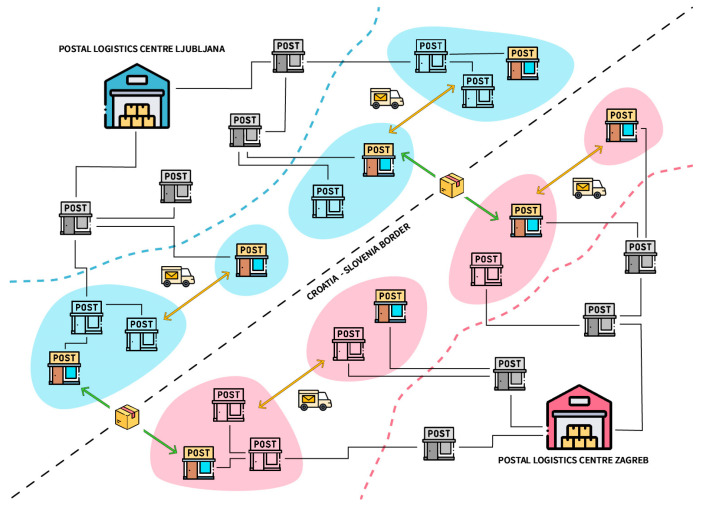
A Cognitive Advisor (CA)-assisted parcel workflow on a cross-border region.

Figure 2 shows the updated routes/steps applied in the experiment so that parcels can be distributed in two ways: via the post office of exchange or, if more convenient, via direct parcel delivery to the foreign delivery post office. In this modus operandi, the delivery post office takes on the role of the local post office of exchange with all its functions. However, there are some criteria that must be met. Direct delivery must be faster, cheaper, and not deviate too much from the driver’s route. The technology used is the CA, which should determine which parcels are eligible for direct delivery, the best pick-up times, transportation routes, and means of transportation. Operators and drivers should receive all instructions via a handheld device (usually a mobile phone).

By enabling ad hoc events, reducing response time, and dynamically optimizing delivery, parcel delivery supported by CA should have several positive impacts on KPIs, such as:

1. Improved utilization—with the installment of CA, better load factor results can be expected as there will be a continuous process of shipment consolidation.
2. Reduced overall route length—since the current routes between the post office of exchange are fixed and cannot be changed, a significant reduction is seen in route length as CA utilizes the existing infrastructure and fleet near the border.
3. Lower fuel consumption—fuel consumption will be lower as all the shipments near the border will be routed directly to post offices on the other side of the border.
4. Lower overall cost—changing the current fixed shipping route will result in shorter transfer times and shorter distances between the stakeholders involved. Through the intervention of CA, lower costs for maintaining the postal chain between postal operators can be achieved.
5. Better customer experience—CA will significantly impact the internal management of infrastructure, fleet portfolio, and other resources. This installment enables both postal operators to offer new services.

The experiment was designed to test and simulate parcel delivery based on cross-border parcels registered in the created digital system (software and analytical platform). End users participated in focus groups to identify and define KPIs, conduct experiments, and evaluate the final results. The user experience provided by the CA system was studied by the pilot end users through an evaluation of the usability, comprehensibility, and user interface (UI) with user experience (UX) aspects of the system. All drivers cooperated and received the necessary information and notifications. On this basis, the KPIs were measured. 

### 2.3. Digital Representation—The Digital Twin of the Postal Operator

**Figure 3:**
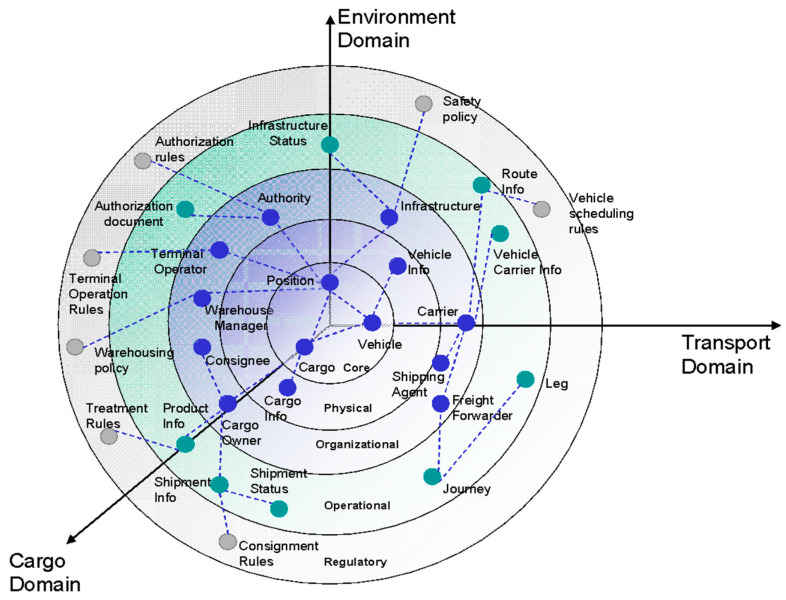
Ontology model for cargo logistics.

First, a new concept was introduced, the Cognitive Logistics Object (CLO), which represents the physical infrastructure: post offices, postal vehicles, and postal logistics centers, etc. A CLO is a virtualized object (similar to a digital twin) or system that participates in the logistics process. It exhibits properties such as autonomy, context awareness, responsiveness, and learning ability [22]. In further modeling, ontology and CLOs representation were used to abstract the entire ecosystem for building a digital representation of postal logistics. For this purpose, the domain-specific ontology model was utilized for entity-relationships representation; see Figure 3 (based on the past research within the FP7 project EURIDICE). The ontological model used focuses on building a services platform, centered on the individual cargo item and on its interaction with the surrounding environment and the user, allowing cargo objects and devices to perform basic interactions on their own and to involve the users’ information systems if needed and when needed.

Most importantly, this enables the establishment of relationships between the different levels of the postal ecosystem: infrastructure objects, parcels, and actuators (people and delivery personnel). CLOs are therefore virtual/digital representations of each infrastructure unit (entity) in the postal operating system, with the defined relationships to other units as the fundamental building blocks for a digital representation of postal operators.

Before processing operational data, the postal system was transformed into a digital representation. First, a representation of the physical infrastructure in the form of an abstract graph was created. It represents each pick-up and drop-off location as a node with edges as the shortest links on the intervening road. This approach allows users to change the digital representation of the system in real time and focus on the data that occur at the location of the event. The graph representation can be changed when a traffic event occurs by changing the connections between nodes. In addition, the size of the graph can be minimized by special methods that use unsupervised ML (clustering) and partitioning the graph into smaller subregions (limiting the complexity of real-time processing and response). The graph represents actual postal locations, and connection between them (length in km). With each event, the graph is changed: with each new site, a new node is added to the graph, and traffic events influence connections between nodes (changed length or connection drops). 

The complete analytical methods were combined into an analytical pipeline to track events and process response recommendations in real time (named the CA). In general parcel workflow management, the CA helps optimize order management by assigning the correct order to the right vehicle and deciding whether that vehicle needs to be rerouted to meet the estimated arrival time. Indeed, it may happen that the courier may receive a new order after he has shipped the scheduled orders for delivery from the sorting center and is on route. In such cases, the courier should accept the new ad hoc order, possibly reschedule deliveries, and change the planned route without affecting the daily order logistics. The CA will also respond to the dynamics of operational constraints and adjust the parcel flow with possible new route recommendations. 

The pilot scenario setup provides a controlled environment for testing. To this end, the domain problems were translated into the main concepts that served as the basis for the data technologies framework. 

### 2.4. Events Processing & Scenarios

To model the system, the logistical processes of Post of Slovenia were observed to represent activities that must be understood, processed, and managed in real time, namely, specific events. The following main classes of events represent the main use cases for data-driven logistics operations in multidimensional process optimization.

1. The most general case is a “**daily plan**” request. In this case, the caller specifies a list of all available vehicles and all parcels. It is assumed that the vehicles are empty. The system returns a plan for each vehicle, calculated by an optimization algorithm.

2. “**Ad hoc requests**” are events related to the parcel delivery process. There are three main categories of ad hoc events (related to the distribution of CLOs):

**a. New parcel pick-up and delivery request;**

**b. Traffic event;**

**c. Vehicle event.**

An ad hoc **parcel pick-up and delivery request** must include a list of available or nearby vehicles with metadata about their current loading and delivery schedules. It also includes information from the post office CLO that initiated the delivery request. The returned routing plans consider the redistribution of parcels for the existing deliveries along with the parcels from the new request.

A **vehicle event** is handled in the case of a vehicle breakdown; the vehicle is functionally a static CLO, whose existing delivery load needs to be redistributed to other vehicles in the vicinity. In the case of a **traffic event**, the caller sends a list of nearby vehicles that may be affected by the event. In this case, the optimization algorithm must recalculate the routing information.

3. A “**cross-border**” delivery request provides a list of nearby vehicles (CLOs), and the resulting routes contain plans for delivery, respecting the region borders by routing deliveries to the CLOs of the exchange nodes.

The use cases have been abstracted into main concepts processed by the CA: daily plan request, ad hoc request, traffic disruption request, cross-border event. Upon receiving the request, the ingest service first categorizes the input based on the specified event and organization metadata provided. The next step is to categorize the requests into one of the above categories.

The main step in the events processing is the construction of the distribution plans for the vehicles. For this purpose, an optimization algorithm was applied, namely, Tabu search algorithm, with formulated Vehicle Routing Problem (VRP). Furthermore, to manage the graph complexity and optimization problem size, the ML techniques were applied, where the graph was partitioned into regions with each region presenting one local optimization. A graph partitioning algorithm was developed, using minimal k-cut approach (where k is the number of clusters). This approach enabled the size of the optimization problem to be reduced to a level appropriate for real-time processing. Though the VRP optimization is an NP-hard problem, using Tabu search heuristics with a combination of ML techniques enabled the requests to be processed in real time, even for an extremely large and dense logistics network (such as the size of European Union region, or large urban areas). A detailed presentation of the algorithmic method can be seen in previous work [22,23].

The functionalities and use cases were tested on real infrastructure testbeds. In this way, the full range of designed events and all levels of complexity could be tested.

### 2.5. Simulations and Data Used

All analytical workflow and algorithms were embedded into the final CA instance, which enabled requests (events) to be received and events to be processed in real time, providing recommendations to drivers for the delivery route. The CA therefore builds an internal digital twin representation of logistics on which it operates optimization algorithms for real-time delivery planning recommendations. Each delivery CLO (driver) was represented via mobile app client and could interact with the CA system for a recommendations update or for reporting an event.

For traffic infrastructure, OSM data were used to create a graph representation. For logistics infrastructure, a sample of real postal locations was used, a sample of distribution vehicles (4 vehicles per postal operator (Post of Slovenia; Post of Croatia), and a representation of historical delivery data for this region (parcel delivery data)). All tested events were simulated by presenting a simulation of the real event data. The daily parcel delivery plan was simulated based on historical samples of daily parcel requests. The ad hoc parcel requests were simulated based on designed events—to include edge cases: different combinations of pick-up locations for delivery, testing different cross-border connections, cyclic route optimization sequences, etc.

The distribution infrastructure event was simulated with a vehicle breakdown, with the driver triggering the event via a mobile app. The traffic event was simulated by (virtually) closing the border and rerouting logistics to other delivery routes and border crossings. All events were tested in the described pilot configurations, with structured KPIs to track the evaluation of the different CA capabilities.

### 2.6. Key Performance Indicators (KPIs): Cross-Country Parcels Deliveries Integration

Specific requirements were defined in the context of cross-country parcel delivery (Table 1). Based on these requirements, the most important KPIs were also defined.

The KPIs were created after special focus groups and workshops with two participating postal operators. Enhanced data sets based on historic data were provided by participating postal operators to calculate the KPIs. Pick-ups and deliveries, routes and itineraries, events, and anything that triggers changes in the pilots’ operational status were simulated based on insights gained from these datasets.

Previous work identified key logistics KPIs that were also used in this pilot. Transportation costs are the total cost of moving goods from the point of production/manufacturing/processing to the point from which the goods are exported or from the point of import to the final destination/distribution or processing facility [24]. Other widely accepted KPIs are the improving utilization factor [25], which as a productivity factor is defined as the ratio of output (e.g., ton-kilometers or vehicle kilometers) to input (e.g., fuel, vehicles, or labor), and utilization: the ratio of actual capacity used to total capacity available (e.g., the amount of space in a container occupied by a load). Other KPIs identified in the literature include fuel consumption/package [26], “on-time delivery in full,” and various operational-level measures, including the ability to provide a daily technical representation, adherence to the developed schedule, ability to avoid complaints, and achieving error-free deliveries [27]. The parallelism of the KPIs derived by the companies with the KPIs identified in the literature is presented in the following sections. Currently, the identified KPIs are mainly related to the logistic aspects and do not cover the IT aspects. In addition to KPIs that are closely related to the end-user context, KPIs that focus on the innovation introduced by CA and the related aspects of ICT performance and usability must also be considered. Since a developed artifact is also part of the KPI evaluation process, we also need to consider a rigorous design evaluation that can rely on many possible techniques, such as analytics, case studies, experiments or simulations [18,28], and naturalistic evaluations [29].

## 3. Results

### 3.1. Development and Monitoring of KPIs

For this pilot, an observational design was used for the evaluation using the field study approach, which was realized by conducting the pilot in the field. More specifically, real data collected from the pilots were used to estimate the various KPIs to demonstrate the effectiveness of the proposed method for monitoring the KPIs. Based on the potential impacts and respective benefits identified during the pilot, a set of initial KPIs was extracted, and these are presented in Table 2. The KPIs are called KA, a combination of K for KPI and A for an (cognitive) advisor.

***KA*1: Load factor.**

The load factor of a vehicle is the ratio of the number of parcels loaded into the vehicle (volumetric weight) divided by the total capacity of the vehicle (maximum possible volume weight). Therefore, the values of the load factor range from 0 (empty vehicle) to 1 (fully loaded vehicle). The main objective of the optimization is to plan the distribution in such a way that the average load factor of the vehicles used in the parcel distribution is maximized (i.e., the maximum utilization of the fleet) (see Equation (1)).

(1) $$KA1=\frac{\frac{number\;of\;parcelson\;v}{capacity\;\left(v\right)}}{N}$$

*v: vehicles;*
*capacity(v): capacity of v in parcels;*
*N: number of vehicles.*

Given the same parcel flow delivery execution, maximizing the load factor results in minimizing the utilization of the distribution vehicles. This has a direct impact on total direct costs and vehicle emissions during parcel distribution. The calculation of this KPI is based on the estimated pay weight of each parcel as a standard measure of the volume of a parcel (see Equation (2)).

(2) $$KA1=\frac{\sum v\;\frac{pw\left(parcels\;on\;v\right)}{pw\;\left(capacity\left(v\right)\right)}}{N}$$

*v: vehicles;*
*capacity(v): capacity of v in parcels;*
*pw: the pay weight of a set of parcels;*
*N: number of vehicles.*

***KA*2: Total route length**

The total route length indicates the total distance traveled by all distribution vehicles in a planned cycle (1 day). One of the main objectives is to minimize the distance traveled for the same number of parcels for delivery. This enables optimization of delivery costs (which are directly correlated to cost/km) and optimal use of delivery resources, as well as reduced environmental impact. The total route length is a key parameter of the primary optimization objective function in all use cases (see Equation (3)).

(3) $$KA2=\sum_{r}distance\left(r\right)$$

*r: routes.*

***KA*3: Fuel consumption**

Fuel consumption is one of the direct costs of parcel delivery and is an important KPI of the optimization objective—considered in the optimization cost function. It represents an estimate of each parcel distribution plan’s cost and environmental efficiency (see Equation (4)).

(4) KA3=∑FC

*FC: fuel costs.*

***KA*4: Response time upon ad hoc orders**

The response time to ad hoc orders shows the performance efficiency of the implemented optimization methods. It is measured by the time it takes CA to process a new event request. This provides an estimate of the efficiency and utilization of the individual CA in different use cases (see Equation (5)).

(5) $$KA4=\frac{\sum_{0}t_{0}}{N}$$

*t*_o_*: response time for* ad hoc *order o;*
*N: number of* ad hoc *orders.*

The response times recorded in the evaluation only include the time CA takes to process the event.

***KA*5: Number of traffic events handled**

*KA*5 was originally developed to measure the number of traffic events handled, which is a ratio of the affected infrastructure connections divided by the number of all infrastructure connections (see Equation (6)).

(6) KA5=N (traffic events handled)

***KA*6: Number of parcel pick-up/delivery events handled**

The number of parcel pick-up/delivery events handled presents the percentage of ad hoc orders relative to total orders in a daily parcel flow that still allows for normal parcel distribution (within daily time constraints). It is calculated as the ratio of ad hoc delivery requests divided by total daily parcel delivery requests (see Equation (7)). The end result is the resilience of CA infrastructure to ad hoc delivery requests—the maximum percentage of daily delivery requests that can be processed as ad hoc requests—for normal operations. For the calculation of this KPI, four delivery vehicles with a load capacity of 100 pay weight each were used.

(7) KA6=N (parcel pickup/delivery events handled)

***KA*7: Total cost**

Minimizing total cost is the primary goal in optimizing parcel delivery. A pick-up/delivery plan consists of a combination of costs related to (i) the total distance covered, (ii) fuel consumption, and (iii) the time required for delivery by the vehicles executing the schedule. The relative total costs are incorporated into the cost function, which is the primary objective function for vehicle scheduling (see Equation (8)). The total cost values indicate the savings during event processing when CA is engaged.

(8) $$KA7=\frac{\left(c\left(fuel\right)+total\;rout\;length\right)}{c\left(p\right)}\;number\;of\;parcels\;delivered$$

*c(p): cost incurred by parameter p*.

### 3.2. Simulation Results

When testing the solution, the Slovenia–Croatia cross-border logistics instance was used. It contained graph partitioning to reduce the size of the graph. This way, it was possible to test all levels of functionalities and capabilities at analytical and performance levels. A region on the border between Slovenia and Croatia was chosen, namely, Brezice–Samobor. This region represents a regional distribution area with 16 post offices (8 per postal operator) and four delivery vehicles (2 per postal operator) with a loading capacity of 200 parcel weights. The post offices were located on both the Slovenian and Croatian sides, including three border crossings (international connections). Each side (Slovenia and Croatia) had a depot node (starting point for vehicles and daily requests). First, the graph construction schemes were validated, as they are the “heart” of the analytical processing. 

To test the services of CA, basic events scenarios were simulated:

- **“Daily plan”**, with four vehicles and parcel delivery requests at all locations.
- **“Ad hoc request”** for ten parcels with different destinations (including deliveries to cross-border locations).
- **“Traffic disruption”** (broken vehicle or traffic event) request with one operational vehicle executing the pick-up request: parcels already loaded on the pick-up vehicle, with different destinations and different pick-up locations.
- **“Cross-border”** event with four vehicles and deliveries for all cross-border post offices included in the delivery plan.

The basic response time to the “DailyPlan” event varied between 25 and 35 s, depending on the scenario and the initialization of the optimization component—which affects the calculation time—for two vehicles. Adding more vehicles increased the time to 35–45 s for three and 45–60 s for four vehicles, respectively.

Similarly, for “Traffic disruption (broken vehicle or traffic event)”, the response time was less than 15–20 s for one operational vehicle included in the event processing and increased to 25–35 s for two vehicles and 35–45 s for three vehicles included in handling broken vehicle parcels pick-ups. For “cross-border” and “ad hoc request” events, the response time was the same as for “DailyPlan”, depending on the number of vehicles included.

The included pilot region was represented as a cluster in the test scenarios, representing a region for distribution. Considering the processing of all events as a single cluster, the response time of CA was fast enough to allow real-time event processing.

In addition, the response to CA recommendations was qualitatively validated using message semantics, including critical situation scenarios such as mixed delivery and pick-up locations in the same route plan or when two different sites have both pick-up and delivery requests, creating a 1-2-1-2 cycle. Using CA, parcels that were assigned to vehicles but not picked up could also be correctly reordered by adding them to existing pick-up orders.

The pilot data set was formed from a week of historical data samples of daily parcel delivery requests in the specific region where the pilot was simulated (daily delivery and intraday delivery parcel requests). These data were extracted from the operational systems used to manage daily postal operations.

The test scenarios were executed in the fully integrated pipeline, including event generation, event routing, and finally, CA event processing resulting in new plan recommendations sent back to CLOs (drivers). The daily requests averaged 150 parcels for each postal operator and included all delivery locations in the pilot. The pay weight of all parcels was normalized and set to 1.0. Ad hoc requests were simulated and included up to 30 new parcels for delivery, with all parcels arriving at the same location. The simulation included different locations for ad hoc requests. The predefined vehicle route plans also included parcels to be picked up at different starting locations. The test scenarios also included the process of driver rejection of a new proposed plan, creation of a new request to the CA, and calculation of a new proposed plan by the CA.

### 3.3. Analysis KPI Improvement

One of the objectives of the pilot experiment was to evaluate the potential for improvement in the cross-border delivery of postal parcel delivery, which was carried out by monitoring the KPIs. The developed KPIs and their improvements in the pilot experiment are shown in Table 3.

Table 3 shows that KPI1 (Load factor) showed the greatest potential for improvement (50.12%). However, some KPIs did not show any potential for improvement. Four out of seven measured KPIs could be improved—in addition to the Load factor, also KPI2 (Route length), KPI3 (Fuel costs), and KPI7 (Total costs). Total costs decreased due to lower fuel consumption, and thus improved fuel economy, and shorter route length, which was one of the key improvements of the CA, allowing for more efficient route planning and adaptation to traffic disruptions.

## 4. Discussion and Conclusions

CA has shown promise for future application in the postal and logistics industry by providing a dynamic approach to managing disruptions in the logistics chain using sophisticated optimization and predictive analytics techniques. From the comparative analysis of the pilot KPIs, it is evident that CA can significantly improve the pilot’s normal business operations. Since there are no real data on the pilot’s previous planning decisions, many simplified assumptions and heuristics for the pilot’s planning processes were adopted to establish the baseline methods for comparison. Nonetheless, the measurements taken as part of the CA assessment are based on actual historical data. From this point of view, the results are sufficiently satisfactory. The encouraging optimization results provided by CA make it possible to provide real-time optimized solutions for any number of CLOs and for various disruption events.

The user experience provided by CA has been well received by the pilot end-users, as evidenced by the in-depth evaluation of the system’s usability, comprehensibility, and UI/UX aspects. CA opens new perspectives by enabling efficient handling of ad hoc events. This alone is sufficient as a general impact to significantly improve the current situation where dynamic, real-time handling of disruption events is poorly supported or not supported at all. Improved KPIs and a higher degree of digitization of delivery processes are also of particular importance in the case of supply disruptions and extraordinary events such as the COVID-19 pandemic, which had a significant impact on the growth of e-commerce in 2020. It is predicted that the share of e-commerce will remain high, with the potential for a further increase in the future, which means that real-time delivery optimization is crucial to handle, manage, and control the increasing number of logistic events with a minimal increase in investment in costly logistics infrastructure. As the Post of Slovenia, Post of Croatia, and numerous other postal operators face the challenge of very low levels of digitization or no digitization at all, a step-by-step improvement process using the existing infrastructure is considered a promising approach.

In this study, an AI/ML framework for the operation and management of parcel distribution for various events in postal logistics was presented. The main objectives of the problem included data sources and digital representation design. The use cases and problems were abstracted into main classes of events to be processed by the developed CA system: daily request, ad hoc request, traffic disruption (e.g., broken vehicle), and cross-border event. Since the Universal Postal Union has created specific standards and rules for digitalization and cross-border deliveries, this sector’s specificities are not considered when such an AI/ML framework and delivery optimization is used in other logistics sectors. As cross-border challenges are recognized by most (all) postal operators, this experiment is a great example of how these challenges can be solved in practice, and extended and implemented on a broad scale. Solutions based on ML may exist [36]; however, they are mainly conceptual [37]. This study, however, is valuable for its potentially broader implementation with the pilot being carried out in a real-life experiment. Its intention is to optimize the cross-border challenges related to distribution, especially for small- and medium-sized cross-border logistics service providers, and this is the most important contribution of this research. The COG-LO project was working on the development of new technical solutions, algorithms that were actually tested in three different logistical settings. However, this paper focuses on one specific pilot experiment involving postal operators in a real application, and similar application experiments are not known to us.

The CA system was successfully designed, developed, and tested based on industry partners’ requirements and use cases. The implementation provides a two-dimensional scaling mitigation mechanism and an analytical pipeline tailored to event classes typical for postal logistics. Nevertheless, some specifically designed logistical KPIs could not be improved (i.e., KPI 4, KPI 5, and KPI 6). However, the potential for utilization is enormous, as based on the existing infrastructure and data technologies, load factor (KPI 1), route length (KPI 2), fuel consumption (KPI 3), and total costs (KPI 7) have been significantly improved. This means that integrating additional infrastructure or data technology improvements could unleash even greater optimization potential for the improved KPIs and all of the currently unimproved ones. Since managing delivery events in real time is one of the main priorities in the digitization process of logistics companies, these types of pilots are going to be expanded and additionally tested under different conditions/environments.

Integrating new and emerging technologies supported by modern business models can significantly improve reliability and resilience. This pilot experiment of digital twin modeling for optimized postal distribution shows the direction of future development of the postal sector, which will also allow it to climb significantly on the Integrated Index for Postal Development and reach the top group (champions) with almost no investments in postal infrastructure, which is usually the most challenging to provide. The most tremendous potential is believed to be in the further expansion also for urban last-mile delivery, which is very demanding and organizationally intensive from the perspective of the working time spent. This study will therefore be further developed for urban delivery to improve last-mile logistics and investigate possible transfer to a multi-company environment for better supply chain management and optimization of multimodal delivery in complex systems with multiple vendors. Thus, there is a growing awareness of the need to improve mobility and transportation and make them more sustainable and competitive by combining traditional technologies with new technologies such as cargo bikes, autonomous vehicles, and drones. In contrast, the complexity of the overall system, characterized by a multitude of actors with conflicting objectives, requires a strategy that harmonizes these actors’ business and operating models [18].

The UPU, as the global umbrella organization for postal organizations, will most likely take up the challenge of developing guidelines and standards for digitized cross-border delivery, which could be a step toward solving the cross-border challenge, especially with regard to delivery processes that are often far from optimal and that can be observed in virtually all postal operators around the world. This pilot scenario could serve as a potential best practice example for solving this problem. Being a country with two million inhabitants, the Slovenian Post and Slovenia are relatively small compared to other postal operators and large countries, so it could take advantage of its small size—high flexibility and easier transition to modern business models. Since the company carries out a large number of cross-border deliveries (due to the country’s small size), it is very well suited to implement tested business models and technologies to improve its KPIs and become one of the best performers. Innovations caused by the rapid development of information and communication technologies and the development of e-government services enable the introduction of electronic postal services as well as the innovation of existing postal services to better meet customer needs and make the postal service more efficient and cost-effective.

The industry is affected by various problems, inefficiencies, and externalities, especially in the last-mile segment, which is why the role of freight transport and parcel delivery has increased. Therefore, there is a growing awareness of the need to improve mobility and transportation models and make them more sustainable and competitive by combining traditional technologies with new technologies such as cargo bikes, autonomous vehicles, and drones along with AI/ML technologies. In contrast, the complexity of the overall system, characterized by a multitude of actors with conflicting goals, requires a strategy that harmonizes the business and operating models of these actors [18]. Therefore, the postal sector sees emerging technologies such as AI, Logistics 4.0, and real-time operations as potentially interesting to increase their competitive advantage, even in the short term [5]. Solutions are evolving toward real-time simulations in a digital environment based on digital twins [5], which can outperform traditional data management and decision-making based on insufficient data, as well as overcome some challenges of uncertain human behavior in complex situations.

## Figures and Tables

**Table 1:** Specific requirements of cross-country parcel delivery.

Focus	Needs
Cross-country	Reduce travel distance for cross-border shipments Cross-country interoperability Reduce transshipment operations Real-time monitoring covering 100% of cargo shipping

**Table 2:** Studied specifics of developed/modified KPIs.

ID	KPI	Developed/Modified from KPI	Measured by/Indicative Data	Current Status	Goal	Derived from
KA1	**Load Factor**	Improved load factor	Ratio between average load and total vehicle capacity (expressed in vehicle km)	Various values (due to different values of cargo). The current value measured in pilot.	Load factor improvement	[30]
KA2	**Total route length**	Shortened XB parcel delivery times in e-commerce	Quality of service standards and route	/	25% improvement	/
KA3	**Fuel consumption**	Reduced XB parcel logistics delivery costs	Cost savings achieved	EMS Sl: up to 50 kg = EUR 75.16 IP Sl: up to 50 kg = EUR 24.02	5% reduction in costs	[26,31,32]
KA4	**Response time upon ad hoc orders**	Swifter response to changing customer needs improving customer satisfaction	Customer satisfaction	Various values (due to the unavailability of XB customer satisfaction measurements, new measures were set)	Improved customer satisfaction	[33]
KA5	**Number of traffic events handled**	Smart parcel/CLO growth	The volume of total smart-parcel services	4.69% (CRO to SLO) 18.70% (SLO to CRO)	30% increase	[34]
KA6	**Number of parcel pick-up/delivery events handled**	Swifter collection and delivery processes, with a more accurate time frame for pick-up and delivery	Quality of service standards and customer satisfaction	Various values (due to the unavailability of measurements of service quality, new measures were set in pilot)	Improved customer satisfaction	[33]
KA7	**Total costs**	Track and trace of parcels through the entire process of delivery, resulting in effective status monitoring both by postal operators and customers.	The success rate of providing track and trace (monitoring) data (real time)	Various values due to lack of monitoring XB track and trace success rate (established track and trace monitoring via existing data, tools, and infrastructure)	Above 95%	[35]

**Table 3:** Results on measured KPIs improvements.

**KA1: Load factor**	**Value** **CA**	**Unit**	**Explanation**	**Improvement**
	65.00	%	The sets of orders are assigned to the vehicles (CLOs) from the social graph, including the existing route plan. The optimal assignment of parcels to the vehicles is found by solving a network optimization model of the fleet with an existing route plan and additional constraints (capacity, pick-up locations, delivery locations, total route length), to create a vehicle dispatch plan. Dispatch plan = min (available vehicles, existing vehicle route plan, vehicle capacity, route lengths)	Yes 50.12%
	**Baseline value**	**Unit**	**Explanation**	
	43.30	%	Average load factor per vehicle by processing ad hoc orders with additional fleet resources without creating new recommendations for rescheduling of the existing vehicle’s route plan.	
**KA2: Total route length**	**Value** **CA**	**Unit**	**Explanation**	**Improvement**
	116	km/vehicle	The sets of orders are assigned to the vehicles (CLOs) from the social graph, including the existing route plan. The optimal assignment of parcels to the vehicles is found by solving a network optimization model of the fleet with an existing route plan and additional constraints (capacity, pick-up locations, delivery locations, total route length), to create a vehicle dispatch plan. Dispatch plan = min (available vehicles, existing vehicle route plan, vehicle capacity, route lengths)	Yes 12.12%
	**Baseline value**	**Unit**	**Explanation**	
	132	km/vehicle	Average total route length by processing ad hoc orders with additional fleet resources without creating new recommendations for rescheduling of the vehicle’s existing route plan.	
**KA3: Fuel Consumption**	**Value** **CA**	**Unit**	**Explanation**	**Improvement**
	9.28	L/vehicle	Same as in KA2. The total route length is multiplied by the average fuel consumption/km. Considering that all vehicles have the same average fuel consumption/km = 0.125 L/km.	Yes 12.12%
	Baseline value	Unit	Explanation	
	**10.56**	L/vehicle	Same as in KA2 multiplied by average fuel consumption/km.	
**KA4: Response time upon ad-hoc orders**	**Value** **CA**	**Unit**	**Explanation**	**Improvement**
	17.2	sec	The time needed to process the CA ad hoc request and create a recommendation response to process negotiation execution by CLOs. The setup with four vehicles in the pilot region.	No
	22.4	sec	The time needed to process the CA ad hoc request and create a recommendation response to process negotiation execution by CLOs. The setup with five vehicles in the pilot region.	
	37.1	sec	The time needed to process the CA ad hoc request and create a recommendation response to process negotiation execution by CLOs. The setup with six vehicles in the pilot region.	
**KA5: Number of traffic events handled**	**Baseline Value**	**Unit**	**Explanation**	**Improvement**
	4.69% CRO to SLO	%	Since the integrated TMS system does not include the Slovenia–Croatia region, the calculations of this KPI were focused only on measuring the number of events related to ad hoc requests, which the CA can handle, i.e., KA6. Thus, KA6 reflects the estimation of distribution elasticity gained by the new logistics system, and KA5 is omitted from the evaluation process.	*n*.a.
	18.70% SLO to CRO			
**KA6: Number of parcel pick-up/delivery events handled**	**Value** **CA**	**Unit** **(DD: daily delivery)**	**Explanation**	**Improvement**
	23 6.38	parcel % DD	Calculated on 90% of total vehicles load capacity loaded with the existing daily plan (360 parcels on daily plan).	No
	36 11.12	parcel % DD	Calculated on 80% of total vehicles load capacity loaded with the existing daily plan (320 parcels on daily plan).	
	62 22.14	parcel % DD	Calculated on 70% of total vehicle load capacity loaded with the existing daily plan (280 parcels on daily plan).	
	104 43.33	parcel % DD	Calculated on 60% of total vehicle load capacity loaded with the existing daily plan (240 parcels on daily plan).	
	177 88.50	parcel % DD	Calculated on 50% of total vehicle load capacity loaded with the existing daily plan (200 parcels on daily plan).	
**KA7: Total costs**	**Value** **CA**	**Unit** **(DD: daily delivery)**	**Explanation**	**Improvement**
	0.596	EUR/parcel	The total costs represent the average costs for delivery vehicles per parcel delivered. The costs represent the costs for traveling the total route length divided by the number of delivered parcels. The total costs represent the average costs for the route traveled per parcel delivered. The KPI is calculated based on values from using CA event management. The average route length per vehicle was 116 km, and the average number of parcels was 175 (150 in the daily plan and 25 in ad hoc requests).	Yes 12.22%
	39.092	EUR/vehicle		
	**Baseline value**	**Unit**	**Explanation**	
	0.679	EUR/parcel	Average costs for route traveled per parcel delivered, calculated based on values for events management without CA. The average route length per vehicle was 132 km, and the average number of parcels was 175 (150 in the daily plan and 25 in ad hoc requests).	
	44.484	EUR/vehicle		

## Data Availability

The next stage of the research is still ongoing, data are not available for sharing yet.
